# Evaluation of a New Bulk Packaging Container for the Ripening of Feta Cheese

**DOI:** 10.3390/foods12112176

**Published:** 2023-05-28

**Authors:** Panagiotis Thodis, Ioanna S. Kosma, Konstantinos Nesseris, Anastasia V. Badeka, Michael G. Kontominas

**Affiliations:** 1 Laboratory of Food Chemistry, Department of Chemistry, University of Ioannina, 45110 Ioannina, Greece; pthodis16@yahoo.com (P.T.); abadeka@uoi.gr (A.V.B.); 2 DODONI S.A. Agricultural Dairy Industry of Epirus, 1 Tagmatarchi Kostaki, Eleousa, 45500 Ioannina, Greece; knesseris@dodoni.eu

**Keywords:** Feta cheese, stainless-steel tank, wooden barrel, tin can container, ripening time, cheese quality

## Abstract

In the present study, the quality characteristics of Feta cheese were investigated as a function of the packaging container (a stainless-steel tank (SST), a wooden barrel (WB), and a tin can (TC)) and ripening time. The results showed that the Feta cheese’s pH, moisture, and lactose decreased, while fat, protein, and salt increased (*p* < 0.05) during ripening with SST and WB, showing similar behaviors versus that of the TC container. For the proteolysis indices, % TN,% WSN, 12% TCA, and 5% PTA showed the highest values (*p* < 0.05) for cheeses packaged in WB, followed by those in SST and TC, with all increasing (*p* < 0.05) during ripening. The most abundant odor-active volatiles were free fatty acids, alcohols, and esters following the order SST > WB > TC on day 60. On day 60, the cheeses packaged in SST and WB showed higher (*p* < 0.05) hardness and fracturability values, as well as aroma scores, compared to those in TC, with both parameter values increasing with the ripening time.

## 1. Introduction

Feta is a white brined cheese, produced in Greece from ewe milk or from mixtures of ewe milk with up to 30% *v*/*v* goat milk. Feta has been approved as a PDO product since 2002 (EU Regulation No. 1829/2002) [1]. As a PDO product, Feta cheese has strict product specifications in terms of its specific composition and method of production within clearly limited production regions in Greece; this provides a substantial added value to the product [1]. Feta production is permitted in specific areas of the Greek mainland and only on the island of Lesvos. Based on this fact, it is quite clear that the composition and the sensory properties of Feta cheese may vary substantially with location but always fall within the legal limits, especially in terms of moisture and fat (moisture ≤ 56%; fat ≥ 43% fat in dry matter, FDM). Furthermore, the animal breed, type of feeding, the flora of the local pasture, the time of milking, agro-climatic conditions, etc., are expected to affect the composition and the sensory properties of milk and consequently those of the resulting cheese [2]. 

During the past few decades, the production of Feta cheese has been industrialized and a number of modifications have been introduced to the cheese-making procedure compared to the basic traditional technology [3]. Basic differences between the traditional and modern technologies include: (i) the former uses rennet produced from lamb and kid abomasa slaughtered before weaning, but this is substituted by synthetic rennet in modern, industrial-scale Feta cheese production; (ii) the former uses traditional yoghourt as a starter culture instead of the mixtures of commercial mesophilic starters that are used in industrial production; (iii) the former uses a milder heat treatment than high-temperature short-time (HTST) pasteurization, which is common practice in the industrial production of cheese, resulting in different microflora of the cheese milk; (iv) in the former, the draining and pre-ripening conditions vary according to weather conditions, as there is usually no temperature control in the ripening rooms of traditionally produced Feta cheese as opposed to strict controls in the ripening conditions of industrially produced cheese; and (v) traditionally produced Feta blocks are packaged in beech or oak wooden barrels and dry salted, whereas industrially produced Feta cheese is packaged in parallelepiped tin cans filled with brine. 

Wood is a porous material, allowing for (1) the gradual inlet of oxygen into the barrel, which interacts with the cheese resulting in the flavor enrichment of the latter, characterizing it as more “sharp”, (2) the exit of a limited amount of moisture from the product, often resulting in a slightly harder texture, and (3) the formation of a biofilm on the internal surface of the barrel, which immobilizes a large number of microorganisms within a thick layer of polysaccharides. Among these microorganisms, lactococci and lactobacilli are of key importance, contributing to the acidification and additional flavor formation of the cheese. On the other hand, tin cans consisting of two-sided tinned steel protect the cheese from the effects of light, oxygen, moisture, odorous compounds, and microorganisms, resulting in Feta cheese with a softer texture and milder flavor. Both wooden barrels and tin containers result in excellent quality Feta cheese, and the choice of either one by consumers is clearly a matter of individual preference [3,4].

Recently, a parallelepiped stainless-steel tank with a cover, either internally lined with beech wood or not, has been experimentally used for convenience purposes, and it is being currently tested as a third bulk ripening/packaging material (in addition to tin can containers and wooden barrels), in which the Feta cheese ripens in brine. 

Based on the above, and given the fact that information on the bulk packaging of Feta cheese during ripening is scarce [5], the objective of the present study was to investigate (1) the effect of the stainless-steel tank, an experimental bulk packaging/ripening container for cheese, in comparison to the conventional wooden barrel and tin can container, on the quality characteristics of Feta cheese and (2) the evolution of Feta’s quality characteristics during ripening using the above experimental packaging container. 

## 2. Materials and Methods

### 2.1. Cheese Preparation

Feta cheese was prepared as part of the commercial production process, in the cheese plant of Dodoni S.A. in Thebes, Greece, in the early spring of 2020 using local ewe and goat milk. Raw ewe and goat milk and a mixture of the two were pasteurized at 72 °C for 15 s; a 70/30 *v*/*v* ratio was used for Feta production, according to the Hellenic Legislation [6]. After cooling to 35 °C, artisanal kid and lamb rennet (Ipirotopoula S.A, Ioannina, Greece) (1:15.000 activity) was added at a rate of 7.5 mL/kg so as to achieve coagulation in about 50 min. The CHOOZIT MT1 LYO 10 DC freeze-dried adjunct starter culture (Chr. Hansen, Denmark) was also added at a rate of 0.75 g/kg. The adjunct culture was composed of *Lactococcus lactis* subsp. *Lactis, Lactococcus lactis* subsp. *Cremoris, Streptococcus thermophilus*, and *Lactobacillus delbrueckii* subsp. *Bulgaricus.* After coagulation, the cheese curd was cut into cubes of dimensions 2 cm × 2 cm × 2 cm and transferred into parallelepiped or cylindrical molds. After draining, the curd from the parallelepiped molds was placed in tin cans (capacity, 17 kg) and stainless-steel tanks lined with wood (capacity 100 kg) (Supplementary Materials Figure S1), while the curd from the cylindrical molds was placed in wooden barrels (capacity, 50 kg). A 70.0 g/kg NaCl solution was added to the cheeses in the tin cans and stainless-steel tanks. Granular NaCl was added to the cheese in the wooden barrels at a concentration of 25.0 g/kg of cheese for a period of 24 h. All containers were then sealed and left to ripen at 16–18 °C for ca. 15 days. Subsequently, the cheeses were transferred into storage/ripening rooms operating at 3–4 °C and kept there for up to 100 days. All cheeses were analyzed for their physico-chemical composition, proteolysis, volatile compounds, and mechanical and sensory properties on days 1, 2, 7, 40, 60, 80, and 100 of storage.

### 2.2. Physico-Chemical Parameter Analyses

The pH of the cheeses was determined using a model WTW (SenTix SP-DIN, 2010) pH meter (Wissenschaftlich-Technische Werkstatten GmbH, Weilheim, Germany). The moisture of the cheeses was determined according to the oven-drying method [7]. Cheese salt content was determined using the Volhard method. The fat content of the cheeses was determined using the Gerber van Gulik method. Cheese protein content, total nitrogen (TN), and soluble nitrogen fractions (water-soluble nitrogen (% WSN), nitrogen soluble in 12% trichloroacetic acid (% TCA-SN), and nitrogen soluble in 5% phosphotungistic acid (% PTA-SN)) were determined according to the Kjeldahl method using Inkjel 1210 M apparatus (Behr Labor-Technik Gmb., Dusseldorf, Germany). Lactose was determined according to Anifantakis [8] as follows: 2 g of cheese were weighed in a 250 mL Erlenmeyer flask; then, 10 mL 0.5 M NaOH and 100 mL deionized water were added, and the mixture was homogenized. The mixture was then transferred to a 200 mL volumetric flask and 10 mL of 10% ZnSO_4_ · 7 H_2_O solution was added to the flask; the flask was filled to the mark. The contents of the flask were then filtered. Then, 1 mL of the filtrate along with 1 mL of 5% phenol were transferred to a new test tube and 5 mL of H_2_SO_4_ (d = 1.84 g/mL) was added dropwise. The test tube was then placed in a water bath at T = 100 °C for 10 min, after which the test tube contents were cooled to 15–20 °C and the absorption of the solution was measured at λ = 490 nm. In parallel, standard solutions of lactose in the range of 0–200 μg/mL were prepared, and their absorption was measured at the same wavelength following the above procedure. The lactose content of cheese was calculated based on the standard curve prepared.

### 2.3. Semi-Quantitative Determination of Volatile Compounds Using Solid Phase Microextraction (SPME)—Gas Chromatography/Mass Spectroscopy (GC/MS)

A 3 g sample of grated cheese, along with 10 μL of an internal standard solution (0.134 mg/mL 4-methyl-2-pentanone), was placed in a 20 mL glass serum vial and sealed with a Teflon-coated septum and an aluminum crimp cap. SPME was performed with a DVB/CAR/PDMS 30/50 μm fiber (Supelco, Bellefonte, PA, USA). 

The vial was then placed in a 50 °C water bath and, after allowing 15 min for equilibration, the fiber was exposed to the vial headspace for 15 min. GC analysis of the volatile compounds adsorbed onto the SPME fiber was carried out on a DB-5MS 60 m × 320 μm i.d. × 1 μm column (Agilent, Santa Clara, CA, USA). An Agilent Technologies 7890A GC System equipped with an Agilent Technologies 5975C MS System detector was used (Wilmington, DE, USA) for the analysis of volatiles. The injector was operated in the split mode (2:1 split ratio) at a temperature of 250 °C. The column was initially maintained at 40 °C for 2 min; it was then heated to 170 °C at a rate of 5 °C/min, then heated to 260 °C at a rate of 10 °C/min and held at 260 °C for 2 min. The flow rate of the helium carrier gas was 1.5 mL/min. The temperature of the transfer line was held constant at 250 °C. The MS conditions were as follows: MS Quadrapole temperature 150 °C; source temperature 230 °C. Acquisition was performed in electron impact (70 eV) mode and the mass/charge was (m/z): 29–300. Peak identification was performed by comparison of the mass spectra of the eluting compounds to those of the Wiley library (Wiley 275, J. Wiley & Sons Ltd., Chichester, England). Retention indices (RI) of volatile compounds were calculated using n-alkane (C8–C20) standards (Fluka, Buchs, Switzerland). All above SPME conditions were the result of optimization carried out in preliminary experiments.

### 2.4. Texture Evaluation

The texture evaluation of cheeses was performed using an Instron Universal Testing Instrument model 4411, (High Wycombe, UK) equipped with a cylindrical plunger 35 mm in diameter. The cheese samples were cut into cube-shaped pieces (with a cube edge of 20 mm) using a stainless-steel cutter. Sampling was carried out on several parts of the cheese body, in order to prepare a representative sample. All measurements were conducted at room temperature (ca. 20 °C). The compression of the sample was set at 70% in one cycle (bite) and the force/time curve was constructed. The operating conditions were (a) a compressive load cell of 5 kN and (b) a crosshead speed of 30 mm/min. Hardness was defined as the peak force (N) during the first compression cycle or “first bite”. Fracturability or brittleness was defined as the force (N) at the first significant break in the curve [9]. 

### 2.5. Sensory Evaluation

The sensory evaluation of the cheeses was carried out according to the IDF protocol [10]. We used a panel of seven experienced judges who consume cheeses regularly. The panel consisted of members of the faculty of the laboratory of Food Chemistry and the R&D Department of the Dodoni S.A. Dairy plant. The panel members were previously trained in evaluation and recognition of the basic tastes of locally made commercial white-brined Feta cheese. The samples were cut into cube-shaped pieces, placed in 3-digit randomly coded plastic cups, and given to the panelists for evaluation. The panelists were asked to eat a cracker biscuit and drink water between their evaluations of samples. The judges evaluated the color/appearance, texture/structure, odor, and taste of the cheeses in separate booths and recorded their scores on evaluation sheets. 

The scoring scale was a 0–5 point scale where:

5 corresponds to very good quality, in accordance with predetermined standards;
4 corresponds to good quality, in accordance with predetermined standards;
3 corresponds to acceptable quality, in accordance with predetermined standards;
2 corresponds to poor quality, in accordance with predetermined standards;
1 corresponds to very poor quality, in accordance with predetermined standards; and
0 corresponds to a product that is unacceptable for consumption.

### 2.6. Statistical Analysis 

Experiments were replicated twice on different occasions. In each trial, 2 cheese samples, 1 from the surface and 1 from the bulk of each cheese block, were collected from all 3 packaging containers (2 replications × 2 samples/replicate × 3 packaging materials × 7 sampling days = 84 samples). All determinations for a given cheese sample were carried out in duplicate (*n* = 2 × 2 = 4). Data were subjected to a two-way analysis of variance (ANOVA) using Minitab 18 software ((Minitab, LLC, State College, PA). Differences between the means of multiple groups were analyzed by two-way ANOVA with Tukey’s multiple range tests. Significant differences were considered for *p* < 0.05. The statistical model used was that of Karakosta et al. [11]. 

## 3. Results and Discussion

### 3.1. Physico-Chemical Parameters of Feta Cheeses

**Figure 1:**
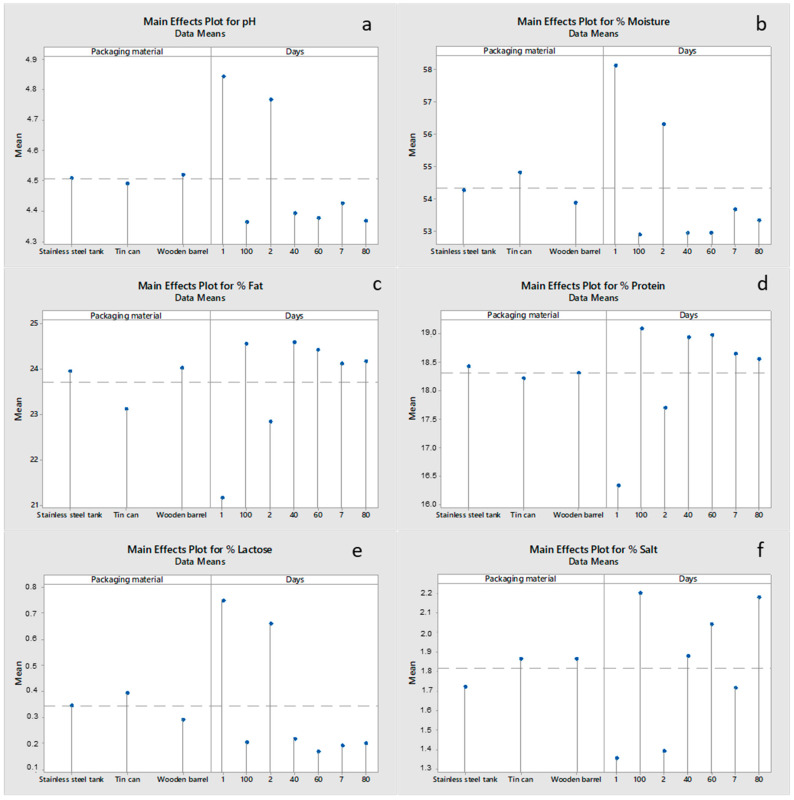
Main effects plots for the physicochemical parameters of (**a**) pH, (**b**) % moisture, (**c**) % fat, (**d**)% protein, (**e**) % lactose, and (**f**) % salt of feta cheese as a function of the packaging material and ripening time.

Changes in the physico-chemical parameter values of cheeses as a function of the packaging material and ripening time are shown in Figure 1. pH (Figure 1a) decreased (*p* < 0.05) with ripening time, ranging between 4.85 (day 1) and 4.34 (day 100), with the packaging material having no effect (*p* > 0.05) for a given sampling day. During ripening, lactose is broken down into lactic acid, resulting in a decreased pH. Acid production is a highly important criterion in the manufacture of cheeses ripened in brine due to the inhibitory effect of lactic acid on the growth of undesirable microorganisms and curd stability during brining [12]. Regarding moisture (Figure 1b), cheeses ripened in the wooden barrel and stainless-steel tank recorded lower (*p* < 0.05) moisture content during the later stages of storage compared to the samples in the tin can. This may be explained by the fact that, of the three packaging materials, only the tin can provided a hermetically sealed environment, resulting in cheeses with a higher moisture content. Moisture content decreased (*p* < 0.05) with ripening time, ranging between 58.2% (day 1) and 52.1 (day 100). This is explained by the fact that, during ripening, moisture is expelled out of the cheese while salt diffuses into the cheese mass [3]. According to Guinee [13], the loss of moisture during cheese ripening is twice as high as the rate of NaCl entering the cheese mass, in proportion to their molecular sizes. Regarding fat (Figure 1c), cheeses ripened in the wooden barrel and stainless-steel tank recorded higher (*p* < 0.05) levels of fat content compared to the sample in the tin can, a fact that can be justified by the lower moisture content of cheeses packaged in wooden barrels and stainless-steel tanks. As moisture dropped during ripening, the fat content increased (*p* < 0.05), ranging between 21.0% (day 1) and 25.5% (day 100). A similar trend regarding the effect of the packaging material and ripening time was shown for the protein content (Figure 1d) as for fat. Protein content ranged between 16.24% (day 1) and 19.54% (day 100). Regarding lactose (Figure 1e), no statistically significant effect (*p* > 0.05) of the packaging material was recorded for a given sampling day. This finding is in agreement with pH values recorded in the study. Lactose decreased (*p* < 0.05) during ripening, ranging between 0.83% (day 1) and 0.20% (day 100) as lactose was converted to lactic acid. Finally, regarding salt content (Figure 1f) cheeses ripened in the wooden barrel and the tin can recorded a higher (*p* < 0.05) salt content compared to the sample in the stainless-steel tank, which is probably related to the different ratios of (brine volume)/(cheese weight) in the three packaging materials used, and possibly to the method of salting (dry salting vs. brine). Salt content increased (*p* < 0.05) during ripening, ranging between 1.05% (day 1) and 2.37% (day 100), as salt slowly diffuses into the cheese mass with time. 

**Figure 2:**
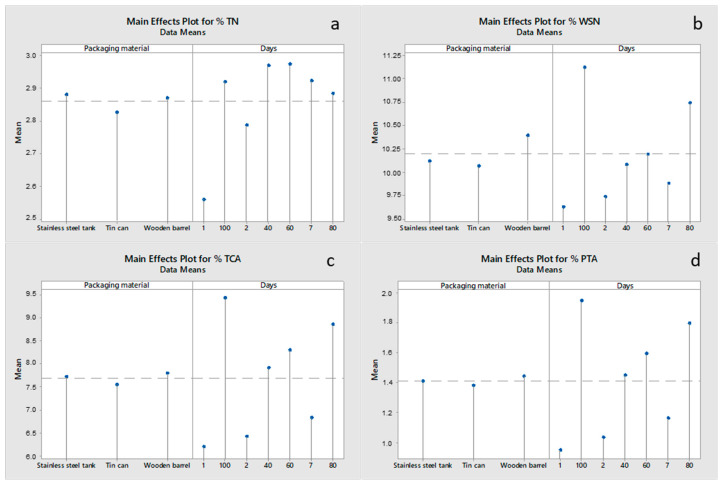
Main effects plots for the proteolysis parameters of (**a**) % TN, (**b**) % WSN, (**c**) % TCA, and (**d**) % PTA of Feta cheese as a function of the packaging material and ripening time.

The extent of proteolysis, the major biochemical event that takes place during cheese ripening, was monitored through the determination of the water-soluble nitrogen (WSN), the nitrogen soluble in 12% trichloroacetic acid (TCA-SN) and the nitrogen soluble in 5% phosphotungstic acid (PTA-SN) (Figure 2). The % ΤΝ (Figure 2a) was affected (*p* < 0.05) by both the packaging material and the ripening time. Cheeses ripened in the wooden barrel and stainless-steel tank recorded a somewhat higher (*p* < 0.05) % TN beginning with day 40 of storage, compared to the sample in the tin can, a finding that may be explained by the lower moisture content of cheeses packaged in the wooden barrel and the stainless-steel tank during the ripening process. As the moisture level dropped in the cheeses during ripening, the % TN increased (*p* < 0.05) from 2.54% (day 1) to 3.00% (day 100). This trend is in agreement with the results for changes in protein content. The % WSN (Figure 2b) was also affected (*p* < 0.05) by both the packaging material and the ripening time. Cheeses ripened in wooden barrels recorded the highest (*p* < 0.05) % WSN content beginning with day 40 of storage, compared to samples ripened in stainless-steel tanks and tin cans. This may be related to the cheeses packaged in wooden barrels having the lowest moisture content compared to the other two packaging materials, resulting in the highest protein content and, logically, in the highest % WSN of cheeses. Regarding the effect of time, as the ripening process proceeded, the % WSN increased (*p* < 0.05) with time due to the gradual breakdown of proteins, ranging from 9.70% (day 1) to 11.51% (day 100). The WSN fraction contains whey proteins, protease-peptone (soluble proteins, peptides, amino acids, amines, urea, ammonia), and low-molecular-weight peptides (<15,000 Dalton molecular mass) derived from casein hydrolysis. According to Rosenberg and Rosenberg [14], the soluble nitrogen compounds are mainly produced by the action of the coagulant. The greatest part of the water-soluble nitrogen consists of nitrogen that is soluble in 12% trichloroacetic acid (TCA), corresponding to medium and small-sized (600–15.000 15,000 Dalton molecular mass) peptides with 2–22 amino acids [3]. According to Fox et al. [15], rennet, bacterial proteinases, and peptidases are responsible for the formation of 12% trichloroacetic acid-soluble nitrogen (TCA-SN). The % TCA (Figure 2c) was affected (*p* < 0.05) by both the packaging material and the ripening time. Cheeses ripened in wooden barrels recorded the highest (*p* < 0.05) % TCA content compared to samples ripened in stainless-steel tanks and tin cans. The explanation for this finding is the same as that for % WSN. Regarding the effect of time, as the ripening process proceeded, the % TCA increased (*p* < 0.05) due to the gradual breakdown of proteins, ranging from 6.21% (day 1) to 9.68% (day 100). Finally, the % PTA followed the same trend as % WSN and % TCA, ranging from 0.95% (day 1) to 1.97% (day 100). The % PTA (Figure 2d) is made up of amino acids and peptides with molecular weights of less than 600 Dalton, which are soluble. The % PTA value is strongly related to the age and flavor intensity of the cheese [3].

The only relevant study in the literature, that of Kondyli et al. [5], investigated the effect of packaging materials (wooden barrels and tin cans) on the physico-chemical parameters of Feta cheese prepared from 100% ewe milk using a combination of (i) a conventional starter culture (*Streptococcus salivarius* subsp. *Thermophilus* and *Lactobacillus L. delbrueckii* subsp. *bulgaricus*) and (ii) artisanal kid and lamb rennet. The above authors reported a lower moisture content and a higher fat content for Feta cheese packaged in wooden barrels compared to that packaged in tin cans, in agreement with results of the present study. However, Feta in barrels had a lower salt content than Feta cheeses stored in tin and a similar protein content after 60 days of storage (the minimum ripening period according to the Greek Codex Alimentarius Athens, Greece [16]). They also reported that packaging material had no effect on proteolysis indices. All physico-chemical parameter values reported in the present study are in general agreement with those of Katsiari et al. [17] and Moatsou et al. [18] for Feta cheese; Sahingil et al. [19] for brined cow cheeses; Zaravela et al. [9] for full- and reduced-fat brined goat cheese; and Plessas et al. [20] for brined ewe cheeses. Differences among the studies, especially those related to proteolysis fractions, may be related to the addition of different rennet, the use of different types of milk, and minor differences in the cheese-making process.

### 3.2. Volatile Compounds of Feta Cheeses

The volatile compounds of the cheeses packaged in the three packaging materials on days 1, 7, 40, 60, 80, and 100 are given in Table 1, Table 2 and Table 3. 

Both the packaging material and the ripening time affected (*p* < 0.05) the volatile profiles of the cheeses. Classes of volatile compounds identified and semi-quantified on day 60 of storage (the minimum ripening period according to the Greek Codex Alimentarius [16]) in order of decreasing concentration were alcohols > free fatty acids > esters > hydrocarbons > aldehydes > ketones > terpenes for cheeses packaged in wooden barrels and stainless-steel tanks; and alcohols ≅ hydrocarbons > aldehydes ≅ esters >free fatty acids > ketones > terpenes for cheeses packaged in tin can containers. Cheeses packaged in stainless-steel tanks and wooden barrels were the richest in volatile compounds compared to those packaged in tin can containers on day 60 of storage (9770.6, 8865.5, and 4666.3 μg/kg respectively). During ripening, the cheeses packaged in wooden barrels and stainless-steel tanks exhibited a large increase in alcohols, free fatty acids, and esters, and a decrease in aldehydes, hydrocarbons and terpenes compared to the first day of storage. During the same period, cheeses packaged in tin can containers exhibited an increase in esters and free fatty acids and a decrease in hydrocarbons, aldehydes, and terpenes. At this point, it should be mentioned that the cheeses packaged in tin can containers had roughly half the amount of volatiles (day 60) than those packaged in the stainless-steel tanks and wooden barrels.

Alcohols were the most abundant volatile compounds in all of the cheeses. Ethanol, the major alcohol in all cheeses, is formed either through lactose fermentation by hetero-fermentative lactic acid bacteria (LAB) or through the metabolism of amino acids or from the reduction of acetaldehyde [5]. Bozoudi et al. [21] also reported that ethanol was the major alcohol in Feta cheese. Primary alcohols, such as butan-1-ol, pentan-1-ol, and heptan-1-ol, are mainly formed by the reduction of their corresponding aldehydes, following a reduction pathway involving alcohol dehydrogenases. When present above their respective threshold levels, aliphatic primary alcohols, such as butan-1-ol and heptan-1-ol, contribute fruity and nutty flavor notes to cheese [22]. According to Meng et al. [23], 3-methyl butanol contributes an alcoholic floral note to cheeses and derives from Leucine via Strecker degradation [24]. Pentan-1-ol, which was identified in all of the cheeses, contributes significantly to the aroma of Greek Feta cheese [5]. The amount of alcohol, in decreasing order (day 60), was 2833.2 > 2570.3 > 1069.3 μg/kg for cheeses packaged in stainless-steel tanks, wooden barrels, and tin can containers, respectively.

Free fatty acids were the second most abundant volatile compounds in Feta cheeses. In general, short- and medium-chain FFAs are the products of lipolysis and contribute to the background flavors of many cheese varieties due to their low perception thresholds and their characteristic flavor notes. Acetic, butanoic, and hexanoic acids were detected in all cheese samples. Acetic acid, which gives the typical flavor of cheeses ripened in brine, is not a product of lipolysis but is mainly produced by other biochemical pathways, probably from the fermentation of lactate or the metabolism of amino acids by bacteria. It contributes to the final flavor of white brined cheese with “pungent” flavor notes and is occasionally detected in brined cheeses [25]. Butanoic acid is responsible for buttery–cheesy flavor notes and hexanoic acid creates sweaty and sometimes pungent flavor notes [26]. When excessive lipolysis occurs in cheeses, the product is characterized by rancid flavors, but cheese varieties such as blue cheese, Italian cheeses, and Greek Feta with a high FFA content are sensorily acceptable [5]. The extensive lipolysis of triglycerides can occur, supported by both microbial and native milk enzymes such as lipases and esterases, and the FFA produced can directly or indirectly contribute to the development of the cheese aroma [26]. Butanoic acid was identified as the most abundant volatile fraction in Feta cheeses [5], while other carboxylic acids, including acetic and hexanoic acids, were found to be the major acids in Turkish white-brined cheeses [27]. The amount of free fatty acids, in decreasing order (day 60), was 2661.9 > 2263.9 > 654.2 μg/kg for cheeses packaged in stainless-steel tanks, wooden barrel, and tin can containers, respectively.

Esters were the third most abundant group of compounds on day 60 of storage. Esters are mainly produced by the enzymatic or chemical reaction of fatty acids with primary alcohols [23] and also by the transesterification of partial glycerides to ethanol [4]. The majority of the esters identified in the present study were ethyl and methyl esters of butanoic, hexanoic, and decanoic acids. Kondyli et al. [5] reported similar differences in ester content for Feta cheeses packaged in wooden barrels and tin can containers. Most of the identified esters have low perception thresholds, with floral and fruity notes, and they probably contribute to the balance of the cheese flavor by minimizing the sharpness imparted by the FFA [28]. Zeng et al. [29] reported that high-quality ewes’ milk cheeses also contained significant amounts of ethyl esters, including ethyl lactate, which are positively correlated with the following sensory attributes: lactic, fruity, floral, and clean cheese flavors. Sahingil et al. [19] reported ethyl esters, including ethyl acetate, ethyl butanoate, ethyl octanoate, and ethyl hexanoate in cow white-brined cheeses. The amount of esters, in decreasing order (day 60), was 2486.8 > 1936.4 > 788.0 μg/kg for cheeses packaged in stainless-steel tanks, wooden barrels, and tin can containers, respectively.

Straight-chain aldehydes, such as propanal, pentanal, hexanal, heptanal, octanal, and nonanal, were detected in the cheeses in different amounts. These aldehydes may be formed via the β-oxidation of unsaturated fatty acids [22]. Aldehydes may make an important contribution to cheese flavor due to their low perception thresholds. Among aldehydes, Sahingil et al. [19] also identified 3-methyl-1-butanal in white-brined cheeses; it is responsible for spicy, cocoa, unripe, apple-like, cheesy, green, malty, and harsh flavors in many cheeses such as Proosdji, Parmesan, Camembert, blue and cheddar. 3-methyl-1-butanal, also detected in cheeses in the present study, is derived from Leucine [30]. The amount of aldehydes, in decreasing order (day 60), was 882.4 > 689.3 > 383.0 μg/kg for cheeses packaged in tin can containers, wooden barrels, and stainless-steel tanks, respectively.

Ketones were not among the major groups of volatile compounds found in the Feta cheeses. In brined cheeses such as Feta, ketones may be produced by the enzymatic decarboxylation of fatty acids by lactic bacteria [28]. Ketones usually play an important role in the flavor of mold-ripened cheeses and they are formed by the action of *Penicillium roqueforti, P. camemberti*, and *Geotrichum candidum* [31]. Among ketones, 3-hydroxy-2-butanone was the most abundant ketone identified in all cheese samples. 3-hydroxy-2-butanone was also determined by Kondyli et al. [5] in Feta cheese and by Sahingil et al. [19] in white-brined cow cheeses. The amount of ketones on day 60 was 202.8 and 83.6 μg/kg for cheeses packaged in wooden barrels and tin can containers, respectively. No. ketones were detected in stainless-steel tanks on day 60 of storage.

A number of miscellaneous compounds were determined in the cheeses (Table 1, Table 2 and Table 3). Of these, two terpenes, α-pinene and limonene, with citrus-like flavors, were identified and semi-quantified. Terpenes are transferred into the milk through the grazing of animals in mountain pastures and are finally detected in the cheese [32]. The linear hydrocarbons detected in the cheeses were probably produced during ripening due to lipid autoxidation [9]. Hexane was present in large amounts in fresh cheeses and significantly decreased (*p* < 0.05) during ripening. Among the numerous linear hydrocarbons, hexane was also identified in Feta cheeses with various geographical origins by Gatzias et al. [33] and Hayaloglou et al. [34] in Tolum cheese. Compounds such as styrene constitute environmental contaminants. The amount of hydrocarbons, in decreasing order (day 60), was 1382.6 > 1174.0 ≅ 1172.2 μg/kg for cheeses packaged in stainless-steel tanks, tin can containers, and wooden barrels respectively.

The presence of aromatic compounds such as alkyl benzenes, which has also been reported in other cheeses, has not been satisfactorily explained, although some authors have postulated that they might be breakdown products of carotene in milk [34]. The amount of terpenes, in decreasing order (day 60), was 30.6 > 23.1 > 14.8 μg/kg for cheeses packaged in wooden barrels, stainless-steel containers, and tin can containers, respectively. 

### 3.3. Textural Parameters of Feta Cheeses

**Figure 3:**
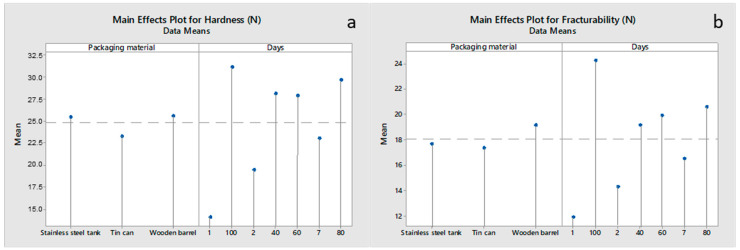
Main effects plots for the textural parameters of the (**a**) hardness and (**b**) fracturability of Feta cheese as a function of the packaging material and ripening time.

The textural parameter values are shown in Figure 3. Cheese hardness (Figure 3a) was affected (*p* < 0.05) by both the packaging material and the ripening time. Cheeses packaged in wooden barrels and stainless-steel tanks (day 60) showed higher (*p* < 0.05) hardness values compared to those packaged in tin can containers. This may be related to the higher loss of moisture in cheeses packaged in wooden barrels and stainless-steel tanks compared to those in the tin cans. Regarding the effect of time, as ripening proceeded, hardness increased (*p* < 0.05) from 13.51 N (day 1) to 32.08 N (day 100). According to McSweeney [35], the texture of cheese undergoes continuous changes during ripening as a consequence of biochemical reactions, i.e., (i) proteolysis, (ii) the decrease in water activity due to the release of water-binding ionic groups, (iii) the redistribution of salt, and (iv) the evaporation of water, as well as changes in pH. On the one hand, during cheese ripening, caseins are hydrolyzed into large and intermediate-sized peptides, which are further transformed into smaller peptides or amino acids by enzymes released from starter and non-starter bacteria. The enzymatic degradation of proteins is thought to reduce cheese hardness [9]. On the other hand, another feature of proteolysis is likely also significant. As each peptide bond is cleaved during proteolysis, two new ionic groups are generated, and each of these competes for the available water in the system. Thus, the water that was previously available for the solvation of the protein chains becomes tied up with the new ionic groups. As a result, the cheese becomes increasingly hard with age and more resistant to deformation. The diffusion of NaCl into the cheese mass also tends to bind the available water, enhancing the above effect [36]. In addition to the above factors, the firmness of cheese tends to increase during ripening, as the loss of moisture from the cheese mass results in an increase in the casein-to-moisture ratio. This often overrides the normal decrease in firmness due to the hydrolysis of the casein matrix [37].

Cheese fracturability/brittleness (Figure 3b) was also affected (*p* < 0.05) by the packaging material and ripening time. Cheeses packaged in wooden barrels showed higher (*p* < 0.05) fracturability on day 40 and beyond compared to those in stainless-steel tanks and tin can containers. This may be attributed to the fact that the latter samples were ripened in the presence of brine, whereby this moisture acted as a plasticizer, resulting in reduced fracturability. Regarding the effect of time, as ripening proceeded, fracturability increased (*p* < 0.05) due to the evolution of protein breakdown, resulting in a weaker casein network. Fracturability ranged from 11.42 N (day 1) to 21.27 N (day 100). 

The present results on texture are in general agreement with those of Topçu and Saldamli [36] for white-brined cow cheeses; Hailu et al. [38] for soft camel cheeses; Zaravela et al. [9] for full-fat and low-fat white-brined goat cheeses; and Basiony and Hassabo [39], who reported the increasing hardness and stress at fracture of for halloumi cheese with ripening time. In contrast to the above findings, Sahingil et al. [19] and Ozbek and Guzeler [40] reported a relatively softer texture for white-brined cow cheeses at the end of ripening, as a result of the hydrolysis of caseins.

### 3.4. Sensory Evaluation of Feta Cheeses

**Figure 4:**
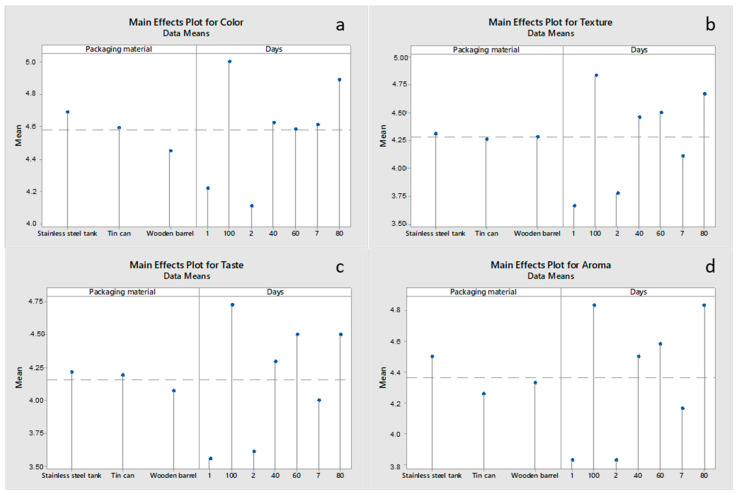
Main effects plots for the sensory parameters of (**a**) color, (**b**) texture, (**c**) taste, and (**d**) aroma of Feta cheese as a function of the packaging material and ripening time.

The sensory parameter values are shown in Figure 4. Color (Figure 4a) was affected (*p* < 0.05) by both the packaging material and the ripening time. During the early stages of ripening, cheeses packaged in stainless-steel tanks had a brighter white color (*p* < 0.05) than those in wooden barrels and tin can containers. Beginning on day 60, the color showed no significant difference (*p* > 0.05) across all the cheese treatments. Regarding the effect of time, color acceptability scores, which are related to the degree of whiteness, increased (*p* < 0.05) in all cheeses from a mean value of 4.2 on day 1, to 4.5 on day 60, to 5.0 on day 100. Such a change may be related to the more pronounced effect of light diffusion on proteins, due to their continuous breakdown during ripening [41]. Zaravela et al. [9] reported no differences in the color of full- and low-fat brined goat cheeses during ripening. Texture (Figure 4b) was not affected by the packaging material but was affected by the ripening time. Texture acceptability scores, which are related to the degree of firmness of the cheese body, increased (*p* < 0.05) from a mean value of 3.7 (day 1) to 4.5 (day 60) to 4.8 (day 100). This finding correlates well with the increase in cheese hardness during ripening (Figure 4a) as a result of salt diffusion into the cheese mass and the synchronous moisture expulsion from the cheese mass (see also Section 3.3). Taste (Figure 4c) was affected by both the packaging material and the ripening time. The taste acceptability scores were the highest for the stainless-steel tank treatment and increased (*p* < 0.05) from a mean value of 3.5 (day 1) to 4.5 (day 60) to 4.7 (day 100). This trend may be explained by enzymatic proteolysis and lipolysis (not determined in the present study) leading to the formation of both volatile and non-volatile flavor-active compounds [35]. This is highly important, since cheeses of up to one year may be consumed without exhibiting over-ripening problems. Finally, aroma (Figure 4d) was affected by both the packaging material and the ripening time. Cheeses packaged in stainless-steel tanks and wooden barrels generally recorded higher (*p* < 0.05) aroma scores during the later stages of storage compared to those in tin can containers. This trend is in agreement with the results on the determination of cheeses volatiles (Section 3.2). Regarding the effect of the ripening time, aroma scores increased (*p* < 0.05) from a mean value of 3.8 (day 1) to 4.6 (day 60) to 4.8 (day 100) as a result of the proteolysis and lipolysis reactions taking place in the cheese.

The present sensory results cannot be compared to the literature as this is the first time a sensory evaluation of Feta cheese has been carried out in relation to the effect of packaging materials on product acceptability. The only published study on the effect of packaging material on quality of Feta cheese, that of Kondyli et al. [5], does not include a product sensory evaluation. With regard to the effect of the ripening time on the quality of Feta cheese, the present results are in general agreement with those of Hamdy et al. [42], who reported no change in color/appearance and an improvement in the flavor of low-fat feta cheese prepared using a mixture of cow and buffalo milk over a 30-day ripening period. Our findings also concur with those of Ozbek and Guzeler [40] for full-fat white cow cheeses, where there was no change in cheese color over a 60-day ripening period, and with those of Basiony and Hassabo [39], who reported an improvement in cheese appearance but no effect on texture in halloumi cheese during a 30-day ripening period. In contrast to the findings of the present study, Sahingil et al. [19] reported no statistically significant changes in sensory properties during the ripening of white-brined cheeses over a period of 120 days. Finally, Moatsou et al. [18] reported that traditional Feta prepared using kid and lamb rennet and packaged in wooden barrels is known to be more flavorful than that prepared in tin cans. This type of rennet was not used in the present study, as the objective of the present study was to determine the potential effect of packaging containers; this required that all other experimental parameters, including the commercial rennet used, were identical.

## 4. Conclusions

In general, wooden barrels and stainless-steel tanks performed better than the tin can containers in relation to Feta cheese’s quality characteristics. During ripening, pH, moisture, and lactose decreased, while fat, protein, and salt increased in the cheese. The proteolysis indices of % TN, % WSN, 12% TCA, and 5% PTA showed the highest values for cheeses packaged in wooden barrels, followed by those in stainless-steel tanks and tin can containers, and the values increased with ripening time. The most abundant volatiles were free fatty acids, alcohols, and esters. The amount of volatile compounds on day 60 followed the order: stainless-steel tank > wooden barrel > tin can container. Cheeses packaged in wooden barrels and stainless-steel tanks (day 60) showed higher (*p* < 0.05) hardness and fracturability values compared to those in tin can containers. Finally, the sensory acceptability of the cheeses followed the order: stainless-steel tank > wooden barrels > tin can containers. In conclusion, the experimental stainless-steel container proved to be the most suitable bulk packaging material for the ripening of Feta cheese, performing better than the conventional tin cans and as well as the wooden barrel, while providing convenience and savings in the use of packaging materials.

## Figures and Tables

**Table 1:** Semi-quantification of the volatile compounds (μg/kg) of the Feta cheese packaged in wooden barrels during the ripening period.

Compound	Rexp *	Rlit **	Days						
			1	2	7	40	60	80	100
Alcohols									
Ethanol	<500	427	80.9 ± 6.7 ^a^	nd	112.2 ± 37.8 ^b^	1500.4 ± 493.3 ^c^	1533.7 ± 105.6 ^c^	1468.5 ± 590.6 ^c^	1538.3 ± 77.8^c^
1-Propanol	538	554	nd	nd	14.5 ± 5.2 ^b^	8.3 ±2.3 ^a^	nd	nd	nd
1-Butanol	649	669	46.7 ± 12.7 ^b^	15.8 ± 7.4 ^a^	1382.9 ± 185.8 ^d^	341.5 ± 62.1 ^c^	362.6 ± 143.0 ^c^	392.3 ± 158.3 ^c^	333.9 ± 35.8 ^c^
1-Butanol, 3-methyl-	726	736	nd	nd	nd	306.7 ± 83.1 ^a^	608.0 ± 170.8 ^b^	1058.9 ± 223.6 ^c^	523.8 ± 209.1 ^b^
2-Methyl butan-1-ol	730	748	nd	nd	nd	nd	35.1 ± 10.9 ^a^	63.1 ± 10.4 ^b^	64.2 ± 5.6^b^
1-Pentanol	757	765	73.8 ± 28.2 ^b^	21.3 ± 16.9 ^a^	131.3 ± 27.5 ^c^	48.5 ± 12.0 ^b^	30.9 ± 7.0 ^a^	32.6 ± 6.4 ^a^	nd
1-Hexanol	859	862	34.4 ± 15.5	nd	nd	nd	nd	nd	nd
Subtotal			235.8 ± 17.1	37.1 ± 13.0	1640.9 ± 95.8	2205.4 ± 225.5	2570.3 ± 110.4	3015.4 ± 291.2	2460.2 ± 113.0
Ketones									
2,3-Butanedione	574	584	nd	17.0 ± 9.5	nd	nd	nd	nd	nd
2-Butanone, 3-hydroxy-	703	717	253.6 ± 39.9 ^c^	164.5 b ± 85.0 ^c^	103.8 ± 10.6 ^b^	48.9 ± 14.6 ^a^	190.4 ± 48.0 ^c^	31.8 ± 5.0 ^a^	28.4 ± 9.2 ^a^
2-Heptanone	881	888	nd	nd	nd	nd	12.4 ± 2.4	nd	nd
Subtotal			253.6 ± 39.9	181.5 ± 60.5	103.8 ± 10.6	48.9 ± 14.6	202.8 ± 34.0	31.8 ± 5.0	28.4 ± 9.2
Aldehydes									
Butanal, 3-methyl-	645	650	nd	nd	nd	8.3 ± 4.4	nd	nd	nd
Hexanal	793	810	886.9 ± 376.8 ^c^	799.6 ± 262.5 ^c^	459.5 ± 143.9 ^b^	689.8 ± 254.4 ^c^	492.3 ± 42.9 ^b^	193.3 ± 71.4 ^a^	225.7 ± 23.7 ^a^
2-Hexenal	848	854	26.2 ± 12.7 ^b^	24.8 ± 11.6 ^b^	6.6 ± 1.4 ^a^	28.4 ± 5.8 ^b^	4.3 ± 1.5 ^a^	nd	nd
Pentanal	690	695	59.7 ± 16.5 ^b^	43.6 ± 8.2 ^b^	17.2 ± 2.8 ^a^	nd	nd	nd	nd
n-Heptanal	895	899	478.0 ± 226.0 ^d^	484.0 ± 190.1 ^d^	269.6 ± 25.3 ^c^	359.9 ± 147.1 ^c,d^	192.7 ± 33.3 ^b^	122.1 ± 28.0 ^a^	88.6 ± 16.3 ^a^
Octanal	997	1002	14.2 ± 4.6	nd	nd	nd	nd	nd	nd
Nonanal	1098	1104	nd	32.5 ± 8.2	nd	nd	nd	nd	nd
Subtotal			1465.0 ± 196.7	1384.5 ± 145.1	752.9 ± 73.1	1086.4 ± 147.0	689.3 ± 19.2	315.4 ± 54.2	314.3 ± 20.3
Esters									
Acetic acid, methyl ester	515	522	nd	nd	nd	nd	49.3 ± 12.8 ^b^	27.9 ± 8.3 ^a^	18.7 ± 3.3 ^a^
Acetic acid, ethyl ester	601	614	nd	nd	nd	172.9 ± 63.6 ^a^	285.0 ± 32.6 ^b^	228.1 ± 98.9 ^a,b^	196.4 ± 55.6 ^a^
Butanoic acid, methyl ester	713	735	440.1 ± 91.2 ^a^	403.9 ± 78.0 ^a^	548.0 ± 164.2 ^b^	415.5 ± 116.3 ^a^	627.0 ± 96.6 ^b^	892.6 ± 150.8 ^c^	333.0 ± 98.6 ^a^
Butanoic acid, ethyl ester	791	798	108.3 ± 36.0 ^a^	nd	nd	355.0 ± 107.6 ^c^	171.5 ± 24.4 ^b^	256.0 ± 90.2 ^c^	284.7 ± 70.5 ^c^
Hexanoic acid, methyl ester	791	798	368.3 ± 51.8 ^a^	518.2 ± 28.7 ^b^	477.4 ± 226.3 ^b^	654.9 ± 127.0 ^c^	608.9 ± 52.0 ^c^	639.3 ± 257.7 ^c^	347.0 ± 125.8 ^a^
Butanoic acid, 2-methylpropyl ester	945	953	nd	nd	nd	6.2 ± 1.7	nd	nd	nd
Hexanoic acid, ethyl ester	987	996	nd	nd	nd	223.4 ± 27.3 ^b^	143.1 ± 37.8 ^a^	230.5 ± 81.1 ^b^	248.5 ± 94.0 ^b^
Octanoic acid, ethyl ester	1184	1193	nd	nd	nd	31.0 ± 6.0 ^b^	nd	18.2 ± 3.5 ^a^	41.9 ± 18.8 ^b^
Decanoic acid, methyl ester	1318	1324	53.9 ± 13.7 ^a^	123.1 ± 29.5 ^b^	75.7 ± 22.6 ^a,b^	100.9 ± 18.0 ^b^	51.6 ± 11.2 ^a^	79.5 ± 19.7 ^a,b^	46.5 ± 12.5 ^a^
Subtotal			970.6 ± 55.9	1045.2 ± 50.9	1101.1 ± 162.0	1959.8 ± 76.1	1936.4 ± 46.9	2372.1 ± 119.4	1516.7 ± 73.3
Hydrocarbons									
Cyclopentane	549	563	34.0 ± 3.1 ^b^	14.8 ± 5.7 ^a^	14.6 ± 5.2 ^a^	nd	nd	23.1 ± 10.0 ^a,b^	9.2 ± 1.0 ^a^
Pentane, 3-methyl-	564	570	12.3 ± 1.7 ^b^	5.1 ± 0.8 ^a^	5.1 ± 1.5 ^a^	nd	nd	nd	nd
Hexane	581	600	4501.2 ± 311.1 ^e^	3634.8 ± 1220.0 ^d,e^	2816.5 ± 1106.9 ^d^	1015.5 ± 356.9 ^b,c^	1165.4 ± 259.8 ^c^	683.9 ± 105.4 ^b^	114.5 ± 21.1 ^a^
Cyclopentane, methyl-	618	635	32.0 ± 2.2 ^b^	12.6 ± 2.9 ^a^	25.8 ± 12.5 ^a,b^	nd	nd	nd	nd
Heptane	692	700	nd	nd	nd	144.4 ± 27.7 ^b^	nd	nd	12.6 ± 2.2 ^a^
Benzene, ethyl-	861	856	nd	nd	nd	30.0 ± 3.7	nd	nd	nd
Styrene	892	895	55.7 ± 10.6 ^c^	nd	10.3 ± 1.9 ^b^	57.1 ± 14.0 ^c^	7.1 ± 1.2 ^a^	11.0 ± 1.1 ^b^	nd
Benzene, 1-methyl	1026	1041	8.1 ± 0.9 ^c^	2.6 ± 0.5 ^a^	5.9 ± 1.2 ^b^	37.7 ± 5.0 ^d^	nd	nd	nd
Subtotal			4643.3 ± 127.1	3669.9 ± 545.6	2878.2 ± 451.9	1284.7 ± 160.2	1172.2 ± 183.7	718.0 ± 61.1	136.3 ± 60.9
Acids									
Acetic acid	577	595	4.0 ± 0.9 ^a^	nd	nd	15.8 ± 2.3 ^b^	nd	nd	nd
Butanoic acid	780	784	24.1 ± 11.8 ^a^	395.9 ± 27.9 ^c^	2383.7 ± 1246.2 ^d^	2273.1 ± 731.4 ^d^	2153.2 ± 624.1 ^d^	218.6 ± 78.7 ^b^	1514.1 ± 336.8 ^d^
Hexanoic acid	955	970	nd	nd	nd	128.9 ± 23.2 ^b^	110.7 ± 9.0 ^b^	36.5 ± 7.0 ^a^	nd
Subtotal			28.1 ± 8.4	395.9 ± 27.9	2383.7 ± 1246.2	2417.8 ± 422.5	2263.9 ± 441.5	255.1 ± 55.9	1514.1 ± 336.8
Terpenes									
a-Pinene	937	943	16.5 ± 3.0 ^a^	33.3 ± 10.2 ^b^	23.7 ± 11.6 ^a,b^	31.1 ± 2.9 ^b^	30.6 ± 4.6 ^b^	37.7 ± 14.5 ^b^	30.5 ± 14.3 ^b^
dl-Limonene	1032	1039	22.6 ± 1.2 ^c^	3.7 ± 0.4 ^a^	7.6 ± 1.3 ^b^	47.2 ± 8.4 ^d^	nd	nd	nd
Subtotal			39.1 ± 2.3	37.0 ± 7.2	31.3 ± 8.2	78.3 ± 6.3	30.6 ± 4.6	37.7 ± 14.5	30.5 ± 14.3
Furans									
Furan, 2-ethyl-	692	706	nd	13.8 ± 3.8	nd	nd	nd	nd	nd
Furan, 2-pentyl-	985	998	nd	2.6 ± 0.5	nd	nd	nd	nd	nd
Subtotal			-	16.4 ± 2.7	-	-	-	-	-
TOTAL			7635.5 ± 112.9	6767.5 ± 270.5	8891.9 ± 372.6	9081.3 ± 193.8	8865.8 ± 156.5	6745.5 ± 159.8	6000.5 ± 102.2

* Experimental retention indices values based on the calculations using the standard mixture of alkanes; ** Retention indices of the identified compounds according to the literature data cited in the NIST MS library; nd: not determined; ^a,b,c …^ different superscripts in a row indicate statistically significant differences, *p* < 0.05.

**Table 2:** Semi-quantification of the volatile compounds (μg/kg) of the Feta cheese packaged in tin cans during the ripening period.

Compound	Rexp *	Rlit **	Days						
			1	2	7	40	60	80	100
Alcohols									
Ethanol	<500	427	80.3 ± 16.1 ^a^	53.3 ± 16.5 ^a^	120.3 ± 24.7 ^b^	291.3 ± 67.0 ^c^	957.8 ± 211.7 ^d^	1070.9 ± 194.1 ^d^	1024.7 ± 388.1 ^d^
1-Butanol	650	669	34.5 ± 5.2 ^a^	40.2 ± 6.0 ^a^	44.2 ± 11.7 ^a^	nd	nd	34.6 ± 3.3 ^a^	nd
1-Butanol, 3-methyl-	726	736	nd	nd	47.9 ± 8.0 ^b^	20.9 ± 6.2 ^a^	90.5 ± 8.4 ^c^	509.1 ± 128.0 ^d^	365.2 ± 150.4 ^d^
1-Pentanol	757	765	512.2 ± 163.3 ^c^	60.5 ± 16.3 ^b^	17.4 ± 3.1 ^a^	nd	21.0 ± 6.5 ^a^	nd	nd
1-Hexanol	859	862	694.9 ± 142.8 ^b^	72.1 ± 7.0 ^a^	nd	nd	nd	nd	nd
Subtotal			1321.9 ± 108.8	226.1 ± 12.5	229.8 ± 14.3	312.2 ± 47.6	1069.3 ± 122.4	1614.6 ± 134.2	1389.9 ± 194.3
Ketones									
2,3-Butanedione	573	584	15.6 ± 5.7 ^b^	nd	nd	nd	nd	6.6 ± 1.0 ^a^	nd
2-Butanone, 3-hydroxy-	702	717	10.6 ± 1.8 ^a^	227.4 ± 112.4 ^d^	92.2 ± 13.8 ^c^	nd	72.0 ± 13.5 ^c^	49.9 ± 4.4 ^b^	38.0 ± 6.8 ^b^
2-Heptanone	881	888	72.6 ± 9.6 _c_	7.7 ± 3.3 ^a^	nd	nd	11.6 ± 2.0 ^a^	28.5 ± 6.2 ^b^	nd
Subtotal			98.8 ± 6.5	235.1 ± 79.5	92.2 ± 13.8	-	83.6 ± 9.6	85.0 ± 4.4	38.0 ± 6.8
Aldehydes									
Propanal	<500	537	29.2 ± 5.6 ^a^	39.6 ± 9.6 ^a^	nd	nd	nd	nd	nd
Butanal, 3-methyl-	645	650	nd	nd	nd	nd	nd	8.7 ± 0.8 ^a^	8.3 ± 1.4 ^a^
Pentanal	691	695	77.5 ± 16.3 ^c^	31.4 ± 3.4 ^a^	48.8 ± 4.4 ^b^	nd	nd	nd	69.9 ± 21.7 ^c^
Hexanal	793	810	98.6 ± 8.8 ^a^	1448.7 ± 315.1 ^d^	715.8 ± 306.4 ^c^	420.7 ± 76.1 ^b,c^	593.3 ± 206.9 ^c^	238.8 ± 56.0 ^b^	302.7 ± 97.4 ^b,c^
2-Hexenal	848	854	718.9 ± 71.3 ^d^	64.1 ± 11.5 ^c^	23.9 ± 2.1 ^b^	nd	9.3 ± 1.6 ^a^	nd	nd
n-Heptanal	895	899	139.8 ± 48.1 ^a^	943.5 ± 185.8 ^c^	454.1 ± 188.9 b^c^	304.4 ± 17.0 ^b^	279.8 ± 137.6 ^b^	83.6 ± 27.7 ^a^	121.2 ± 36.5 ^a^
Octanal	997	1002	33.9 ± 17.7 ^a^	98.9 ± 23.3 ^b^	nd	nd	nd	nd	nd
Nonanal	1098	1104	39.9 ± 19.1 ^a^	61.6 ± 5.8 ^b^	33.6 ± 3.0 ^a^	nd	nd	nd	nd
2-Nonenal	1159	1146	139.5 ± 66.7 ^b^	34.3 ± 9.4 ^a^	nd	nd	nd	nd	nd
Subtotal			1277.3 ± 40.2	2722.1 ± 129.8	1276.2 ± 136.1	725.1 ± 55.1	882.4 ± 143.5	331.1 ± 36.1	502.1 ± 53.1
Esters									
Acetic acid, methyl ester	512	522	nd	nd	nd	nd	28.6 ± 9.5 ^a^	31.5 ± 5.5 ^a^	nd
Acetic acid, ethyl ester	601	614	nd	nd	nd	27.8 ± 4.3 ^a^	246.6 ± 61.6 ^c^	284.3 ± 46.0 ^c^	80.8 ± 13.9 ^b^
Butanoic acid, ethyl ester	791	798	105.4 ± 18.2 ^a^	95.8 ± 37.2 ^a^	nd	240.4 ± 134.9 ^b^	95.7 ± 8.2 ^a^	124.1 ± 16.9 ^a^	314.4 ± 84.3 ^b^
Hexanoic acid, methyl ester	914	934	378.4 ± 61.3 ^a^	486.5 ± 109.7 ^a^	336.9 ± 98.5 ^a^	345.9 ± 199.1 ^a^	316.4 ± 150.2 ^a^	454.1 ± 114.8 ^a^	498.6 ± 129.9 ^a^
Hexanoic acid, ethyl ester	987	996	nd	nd	nd	138.9 ± 55.8 ^b^	nd	nd	41.4 ± 7.2 ^a^
Octanoic acid, methyl ester	1115	1125	47.5 ± 6.0 ^a^	192.5 ± 20.1 ^c^	114.6 ± 37.3 ^b^	90.1 ± 44.5 ^b^	80.4 ± 36.7 ^b^	93.5 ± 19.2 ^b^	101.1 ± 26.6 ^b^
Decanoic acid, methyl ester	1320	1324	83.9 ± 7.6 ^b^	146.6 ± 27.8 ^c^	69.1 ± 37.3 ^b^	56.6 ± 7.3 ^b^	20.3 ± 5.1 ^a^	43.1 ± 17.7 ^a,b^	47.6 ± 27.6 ^a,b^
Subtotal			615.1 ± 32.3	921.4 ± 60.4	520.6 ± 64.5	899.7 ± 102.5	788.0 ± 68.2	1030.6 ± 52.1	1083.9 ± 65.4
Hydrocarbons									
Cyclopentane	550	563	6.1 ± 1.0 ^a^	23.5 ± 2.5 ^c^	11.8 ± 2.0 ^b^	nd	41.1 ± 3.5 ^d^	56.2 ± 4.8 ^d^	48.3 ± 10.2 ^c,d^
Pentane, 3-methyl-	566	570	38.2 ± 5.1 ^c^	120.0 ± 18.4 ^d^	9.5 ± 3.9 ^b^	nd	nd	nd	2.0 ± 0.3 ^a^
Hexane	583	600	3.9 ± 0.6 ^a^	3712.8 ± 1273.8 ^e^	3621.1 ± 1083.6 ^e^	39.3 ± 6.8 ^b^	1121.6 ± 541.2 ^c^	1216.6 ± 210.0 ^c^	2006.4 ± 909.2 ^d^
Cyclopentane, methyl-	619	635	4499.2 ± 310.6 ^b^	22.9 ± 5.8 ^a^	24.1 ± 2.1 ^a^	nd	nd	nd	nd
Benzene, ethyl-	861	856	50.3 ± 4.3 ^b^	nd	nd	12.7 ± 2.1 ^a^	nd	nd	nd
Styrene	892	895	7.7 ± 1.3 ^a^	135.2 ± 50.0 ^d^	34.8 ± 17.0 ^c^	43.3 ± 14.0 ^c^	11.3 ± 1.9 ^a^	nd	18.1 ± 1.6 ^b^
Benzene, methyl	1026	1041	53.8 ± 6.1 ^c^	15.6 ± 6.1 ^b^	4.0 ± 0.7 ^a^	26.0 ± 12.6 ^b^	nd	nd	4.5 ± 0.8 ^a^
Subtotal			4659.2 ± 117.4	4030.0 ± 520.5	3705.3 ± 442.4	121.3 ± 10.0	1174.0 ± 312.5	1272.8 ± 148.5	2079.3 ± 406.6
Acids									
Acetic acid	577	595	15.7 ± 2.7 ^a^	40.2 ± 4.7 ^b^	nd	nd	nd	nd	nd
Butanoic acid	780	784	nd	nd	1177.5 ± 618.1 ^c^	778.0 ± 47.1 ^b^	565.5 ± 104.7 ^a^	434.4 ± 166.5 ^a^	1081.3 ± 322.6 ^c^
Hexanoic acid	961	970	nd	nd	135.1 ± 15.9 ^b^	10.5 ± 0.8 ^a^	88.7 ± 10.5 ^b^	7.2 ± 1.2 ^a^	106.3 ± 24.6 ^b^
Subtotal			15.7 ± 2.7	40.2 ± 4.7	1312.6 ± 437.2	788.5 ± 33.3	654.2 ± 74.4	441.6 ± 117.7	1187.6 ± 228.8
Terpenes									
α-Pinene	937	943	366.4 ± 110.6 ^c^	17.8 ± 4.6 ^a,b^	13.8 ± 5.2 ^a^	16.0 ± 5.9 ^a,b^	14.8 ± 3.2 ^a^	20.3 ± 1.5 ^b^	20.3 ± 4.5 ^b^
dl-Limonene	1032	1039	39.6 ± 10.8 ^c^	28.7 ± 3.7 ^c^	12.9 ± 5.6 ^b^	34.4 ± 14.4 ^c^	nd	nd	5.6 ± 0.9 ^a^
Subtotal			406.0 ± 78.6	46.5 ± 4.2	26.7 ± 5.4	50.4 ± 11.0	14.8 ± 3.2	20.3 ± 1.5	25.9 ± 3.2
Furans									
Furan, 2-ethyl-	985	993	83.1 ± 33.6 ^b^	10.6 ± 1.8 ^a^	9.2 a ± 1.6 ^a^	nd	nd	nd	nd
Furan, 2-pentyl-	985	993	3.8 ± 0.6 ^a^	63.6 ± 10.6 ^b^	nd	nd	nd	nd	nd
Subtotal			86.9 ± 23.8	74.2 ± 7.6	9.2 ± 1.6	-	-	-	-
TOTAL			8481.0 ± 75.0	8295.6 ± 248.3	7172.6 ± 266.1	2897.2 ± 65.2	4666.3 ± 148.1	4796.0 ± 85.5	6306.7 ± 227.8

* Experimental retention indices values based on the calculations using the standard mixture of alkanes; ** Retention indices of the identified compounds according to the literature data cited in the NIST MS library; nd: not determined; ^a,b,c …^ different superscripts in a row indicate statistically significant differences, *p* < 0.05.

**Table 3:** Semi-quantification of the volatile compounds (μg/kg) of the Feta cheese packaged in stainless-steel tanks during the ripening period.

Compound	Rexp *	Rlit **	Days						
			1	2	7	40	60	80	100
Alcohols									
Ethanol	<500	427	72.2 ± 6.4 ^a^	nd	129.5 ± 11.2 ^b^	1057.5 ±200.4 ^c^	1745.6 ± 179.2 ^d^	1535.6 ± 272.0 ^d^	1420.9 ± 118.3 ^d^
1-Propanol	538	554	nd	nd	nd	nd	53.8 ± 6.0 ^a,b^	68.9 ± 13.4 ^b^	38.6 ± 9.7 ^a^
3-Methyl-1-butanol	726	736	nd	nd	51.8 ± 8.9 ^a^	282.6 ± 24.6 ^b^	736.3 ± 135.6 ^c^	530.7 ± 101.2 ^c^	218.5 ± 49.5 ^b^
1-Butanol	649	669	41.6 ± 3.7 ^a^	nd	130.8 ± 13.7 ^b^	nd	194.2 ± 101.8 ^c^	nd	190.1 ± 28.2 ^c^
1-Pentanol	757	765	34.8 ± 3.0 ^a^	26.3 ± 4.5 ^a^	27.8 ± 4.8 ^a^	nd	nd	nd	nd
1-Hexanol	859	862	nd	nd	nd	nd	103.1 ± 48.6	nd	nd
Subtotal			148.6 ± 4.6	26.3 ± 4.5	339.9 ± 10.2	1340.1 ± 142.8	2833.2 ± 110.7	2135.2 ± 167.7	1868.1 ± 65.8
Ketones									
2-Butanone, 3-hydroxy-	703	717	415.4 ± 69.7 ^d^	167.4 ± 28.9 ^c^	46.7 ± 4.4 ^a^	nd	nd	75.6 ± 5.5 ^b^	nd
2-Heptanone	881	888	nd	nd	8.0 ± 3.9	nd	nd	nd	nd
Subtotal			415.4 ± 69.7	167.4 ± 28.9	54.7 ± 4.1	-	-	75.6 ± 5.5	-
Aldehydes									
Propanal	<500	537	nd	21.1 ± 3.6	nd	nd	nd	nd	nd
Hexanal	793	810	743.6 ± 126.6 ^c^	715.9 ± 272.7 ^c^	692.2 ± 279.9 ^c^	107.3 ± 45.9 ^a^	274.9 ± 90.2 ^b^	240.9 ± 16.3 ^b^	160.0 ± 14.9 ^a^
2-Hexenal	848	854	8.3 ± 1.4 ^a^	15.6 ± 2.7 ^b^	21.8 ± 2.2 ^c^	nd	nd	nd	nd
n-Heptanal	895	899	367.9 ± 112.4 ^b^	442.5 ± 129.5 ^b^	398.4 ± 177.6 ^b^	70.0 ± 6.0 ^a^	108.1 ± 50.8 ^a^	75.7 ± 9.8 ^a^	66.7 ± 6.2 ^a^
3-Methyl- butanal	645	650	nd	nd	nd	19.8 ± 3.4 ^a^	nd	414.3 ± 21.0 ^b^	nd
Pentanal	690	695	35.3 ± 3.0 ^b^	39.7 ± 4.6 ^b^	34.2 ± 5.9 ^b^	97.4 ± 9.1 ^c^	nd	nd	25.9 ± 4.4 ^a^
Nonanal	1098	1104	nd	nd	19.0 ± 3.2	nd	nd	nd	nd
Subtotal			1155.1 ± 84.7	1234.8 ± 135.0	1165.8 ± 148.3	294.5 ± 23.6	383.0 ± 73.2	730.9 ± 16.4	252.6 ± 9.7
Esters									
Butanoic acid, methyl ester	713	735	866.0 ± 254.9 ^b^	396.6 ± 137.6 ^a^	360.5 ± 161.2 ^a^	280.3 ± 109.2 ^a^	587.5 ± 218.2 ^b^	588.8 ± 118.1 ^b^	418.0 ± 140.4 ^a^
Butanoic acid, ethyl ester	791	798	80.4 ± 6.9 ^a^	146.0 ± 56.9 ^b^	nd	375.4 ± 136.0 ^c^	254.5 ± 104.7 ^b,c^	221.3 ± 51.5 ^b,c^	208.5 ± 99.6 ^b,c^
Butanoic acid, 2-methylpropyl ester	945	953	nd	nd	nd	9.0 ± 1.5	nd	nd	nd
Butanoic acid, 3-methyl-, ethyl ester	844	842	nd	nd	nd	10.8 ± 1.8 ^a^	nd	108.0 ± 44.4 ^c^	42.3 ± 7.3 ^b^
Acetic acid, methyl ester	515	522	nd	nd	nd	nd	106.1 ± 49.6 ^b^	nd	43.2 ± 4.4 ^a^
Acetic acid, ethyl ester	601	614	nd	nd	nd	146.8 ± 53.5 ^a^	288.2 ± 26.5 ^b^	294.2 ± 31.2 ^b^	192.2 ± 43.4 ^a^
1-Butanol, 3-methyl-, acetate	866	881	nd	nd	nd	nd	24.2 ± 4.1	nd	nd
Hexanoic acid, methyl ester	914	934	570.5 ± 215.5 ^ab^	352.0 ± 150.7 ^a^	469.5 ± 174.9 ^ab^	416.5 ± 45.5 ^a^	684.5 ± 192.7 ^b^	671.2 ± 119.3 ^b^	332.5 ± 45.0 ^a^
Hexanoic acid, ethyl ester	987	996	nd	nd	nd	198.1 ± 19.0 ^b^	262.8 ± 78.4 ^b^	140.9 ± 49.0 ^ab^	117.8 ± 32.3 ^a^
Octanoic acid, methyl ester	1113	1125	130.4 ± 35.0 ^b^	98.0 ± 24.5 ^a^	139.2 ± 26.5 ^b^	85.7 ± 5.6 ^a^	182.5 ± 77.4 ^b^	154.0 ± 22.0 ^b^	86.3 ± 4.0 ^a^
Octanoic acid, ethyl ester	1184	1193	nd	nd	nd	nd	5.5 ± 0.9	nd	nd
Decanoic acid, methyl ester	1318	1324	61.1 ± 28.4 ^a^	100.3 ± 23.5 ^b^	120.3 ± 11.7 ^b^	28.4 ± 2.9 ^a^	91.0 ± 9.4 ^b^	81.3 ± 3.1 ^b^	33.7 ± 3.0 ^a^
Subtotal			1708.4 ± 150.7	1092.9 ± 96.0	1089.5 ± 119.8	1551.0 ± 63.0	2486.8 ± 105.4	2259.7 ± 67.7	1474.5 ± 62.1
Hydrocarbons									
Cyclopentane	549	563	39.5 ± 4.1 ^c^	13.9 ± 1.3 ^b^	11.8 ± 2.0 ^b^	nd	65.8 ± 13.2 ^d^	61.7 ± 4.0 ^d^	6.2 ± 1.0 ^a^
3-Methyl-pentane	564	570	14.3 ± 0.8 ^b^	3.3 ± 0.6 ^a^	nd	nd	nd	nd	nd
Hexane	581	600	4160.6 ± 1201.4 ^c^	2766.4 ± 1079.6 ^c^	4010.7 ± 1237.7 ^c^	1378.5 ± 55.6 ^b^	1290.8 ± 238.2 ^a,b^	1043.5 ± 177.8 ^a^	1563.9 ± 461.5 ^a,b^
Cyclopentane, methyl-	618	635	24.2 ± 2.1 ^b^	10.1 ± 1.7 ^a^	13.6 ± 2.3 ^a^	nd	nd	nd	nd
Styrene	892	895	77.4 ± 29.5 ^c,d^	66.6 ± 5.7 ^d^	nd	49.2 ± 13.2 ^c^	26.0 ± 4.5 ^b^	nd	8.9 ± 1.5 ^a^
Benzene, ethyl	861	856	nd	nd	nd	27.6 ± 2.5	nd	nd	nd
Benzene, methyl	1026	1041	9.2 ± 2.0 ^b^	3.7 ± 0.6 ^a^	nd	34.8 ± 13.8 ^c^	nd	nd	4.1 ± 0.7 ^a^
Subtotal			4325.2 ± 490.6	2864.0 ± 440.7	4036.1 ± 714.6	490.080 ± 29.4	1382.6 ± 137.8	1105.2 ± 125.8	1583.1 ± 230.8
Acids									
Acetic acid	577	595	nd	nd	nd	nd	nd	181.1 ± 13.6 ^a^	158.1 ± 24.2 ^a^
Butanoic acid	780	784	128.6 ± 61.8 a	172.5 ± 50.2 ^a^	1713.6 ± 397.9 ^b^	1214.6 ± 371.2 ^b^	2655.8 ± 833.4 ^c^	3361.3 ± 598.8 ^c^	2097.2 ± 356.7 b^c^
Hexanoic acid	955	970	nd	nd	164.0 ± 32.9 ^d^	40.8 ± 4.8 ^b^	6.1 ± 1.0 ^a^	117.1 ± 8.6 ^c^	545.1 ± 187.4 ^e^
Subtotal			128.6 ± 61.8	172.5 ± 50.2	1877.6 ± 281.7	1255.4 ± 262.5	2661.9 ± 589.3	3659.5 ± 345.8	2800.4 ± 233.0
Terpenes									
α-Pinene	937	943	17.6 ± 3.7 b	173.2 ± 76.5 ^c^	17.1 ± 5.8 ^b^	21.1 ± 5.7 ^b^	23.1 ± 1.2 ^b^	18.9 ± 3.1 ^b^	8.1 ± 0.7 ^a^
dl-Limonene	1032	1039	19.6 ± 2.3 c	17.0 ± 5.4 ^c^	3.9 ± 0.7 ^a^	47.3 ± 18.6 ^d^	nd	nd	5.8 ± 1.0 ^b^
Subtotal			37.2 ± 3.1	190.2 ± 54.2	21.0 ± 4.1	68.4 ± 13.8	23.1 ± 1.2	18.9 ± 3.1	13.9 ± 0.9
Furans									
Furan, 2-ethyl-	692	706	nd	9.1 ± 1.5	nd	nd	nd	nd	nd
Furan, 2-pentyl-	985	998	nd	21.0 ± 2.6	nd	nd	nd	nd	nd
Subtotal			-	30.1 ± 2.1	-	-	-	-	-
TOTAL			7918.5 ± 269.3	5778.2 ± 238.8	8584.4 ± 290.6	5999.5 ± 97.9	9770.4 ± 201.6	9985.0 ± 160.1	7992.6 ± 130.9

* Experimental retention indices values based on the calculations using the standard mixture of alkanes; ** Retention indices of the identified compounds according to the literature data cited in the NIST MS library; nd: not determined; ^a,b,c …^ different superscripts in a row indicate statistically significant differences, *p* < 0.05.

## Data Availability

Data is contained within the article or Supplementary Materials.

## Supplementary Materials

The following supporting information can be downloaded at: https://www.mdpi.com/article/10.3390/foods12112176/s1. Figure S1: (a) wooden barrel, (b) tin can, (c) stainless-steel tank and (d) stainless-steel tank lined with wood for the ripening/bulk packaging of Feta cheese.

## References

[1] European Union (2002). COMMISSION REGULATION (EC) No 1829/2002 of 14 October 2002 amending the Annex to Regulation (EC) No 1107/96 with regard to the name ‘Feta’. Off. J. Eur. Union.

[2] Bravo-Lamas L., Aldai N., Kramer J.K.G., Barron L.J.R. (2018). Case study using commercial dairy sheep flocks: Comparison of the fat nutritional quality of milk produced in mountain and valley farms. LWT.

[3] Moatsou G., Massouras T., Kandarakis I., Anifantakis E. (2002). Evolution of proteolysis during the ripening of traditional Feta cheese. Lait.

[4] Papadopoulou O.S., Argyri A.A., Varzakis E.E., Tassou C.C., Chorianopoulos N.G. (2018). Greek functional Feta cheese: Enhancing quality and safety using a Lactobacillus plantarum strain with probiotic potential. Food Microbiol..

[5] Kondyli E., Pappa E.C., Vlachou A.M. (2012). Effect of package type on the composition and volatile compounds of Feta cheese. Small Rumin. Res..

[6] (1993). Hellenic Legislation Recognition of Feta as Protected Denomination of Origin Cheese.

[7] (2000). Joint Fao/Who Food Standards Programme Codex Committee on Milk and Milk Products.

[8] Anifantakis E. (1992). Methods for the Analysis of Milk and Dairy Products.

[9] Zaravela A., Kontakos S., Badeka A.V., Kontominas M.G. (2021). Effect of adjunct starter culture on the quality of reduced fat, white, brined goat cheese: Part I. Assessment of chemical composition, proteolysis, lipolysis, texture and sensory attributes. Eur. Food Res. Technol..

[10] (2023). Milk and Milk Products—Sensory Analysis—Part 2: Methods for Sensory Evaluation.

[11] Karakosta L.K., Vatavali K.A., Kosma I.S., Badeka A.V., Kontominas M.G. (2022). Combined Effect of Chitosan Coating and Laurel Essential Oil (Laurus nobilis) on the Microbiological, Chemical, and Sensory Attributes of Water Buffalo Meat. Foods.

[12] Coelho M.C., Malcata F.X., Silva C.C.G. (2022). Lactic Acid Bacteria in Raw-Milk Cheeses: From Starter Cultures to Probiotic Functions. Foods.

[13] Guinee T.P. (2004). Salting and the role of salt in cheese. Int. J. Dairy Technol..

[14] Rosenberg M., Rosenberg Y. (2022). Proteolysis during aging of commercial full-fat and reduced-fat Cheddar cheeses of identical chronological age. AIMS Agric. Food.

[15] Fox P.F., Guinee T.P., Cogan T.M., McSweeney P.L.H. (2016). Biochemistry of Cheese Ripening. Fundamentals of Cheese Science.

[16] (2014). Greek Codex Alimentarius Greek Codex for Foods and Beverages.

[17] Katsiari M.C., Voutsinas L.P., Kondyli E., Alichanidis E. (2002). Flavour enhancement of low-fat Feta-type cheese using a commercial adjunct culture. Food Chem..

[18] Moatsou G., Moschopoulou E., Georgala A., Zoidou E., Kandarakis I., Kaminarides S., Anifantakis E. (2004). Effect of artisanal liquid rennet from kids and lambs abomasa on the characteristics of Feta cheese. Food Chem..

[19] Sahingil D., Hayaloglu A.A., Simsek O., Ozer B. (2014). Changes in volatile composition, proteolysis and textural and sensory properties of white-brined cheese: Effects of ripening temperature and adjunct culture. Dairy Sci. Technol..

[20] Plessas S., Ganatsios V., Mantzourani I., Bosnea L. (2021). White brined cheese production by incorporation of a traditional milk-cereal prebiotic matrix with a candidate probiotic bacterial strain. Appl. Sci..

[21] Bozoudi D., Kondyli E., Claps S., Hatzikamari M., Michaelidou A., Biliaderis C.G., Litopoulou-Tzanetaki E. (2018). Compositional characteristics and volatile organic compounds of traditional PDO Feta cheese made in two different mountainous areas of Greece. Int. J. Dairy Technol..

[22] Cuffia F., Bergamini C.V., Wolf I.V., Hynes E.R., Perotti M.C. (2019). Influence of the culture preparation and the addition of an adjunct culture on the ripening profiles of hard cheese. J. Dairy Res..

[23] Meng H.Y., Piccand M., Fuchsmann P., Dubois S., Baumeyer A., Tena Stern M., Von Ah U. (2021). Formation of 3-Methylbutanal and 3-Methylbutan-1-ol Recognized as Malty during Fermentation in Swiss Raclette-Type Cheese, Reconstituted Milk, and de Man, Rogosa, and Sharpe Broth. J. Agric. Food Chem..

[24] Smit G., Smit B.A., Engels W.J.M. (2005). Flavour formation by lactic acid bacteria and biochemical flavour profiling of cheese products. FEMS Microbiol. Rev..

[25] Hayaloglu A.A., Tolu C., Yasar K., Sahingil D. (2013). Volatiles and sensory evaluation of goat milk cheese Gokceada as affected by goat breeds (Gokceada and Turkish Saanen) and starter culture systems during ripening. J. Dairy Sci..

[26] D’Incecco P., Limbo S., Hogenboom J., Rosi V., Gobbi S., Pellegrino L. (2020). Impact of extending hard-cheese ripening: A multiparameter characterization of Parmigiano reggiano cheese ripened up to 50 months. Foods.

[27] Özer B., Kirmaci H.A., Hayaloglu A.A., Akçelik M., Akkoç N. (2011). The effects of incorporating wild-type strains of Lactococcus lactis into Turkish white-brined cheese (Beyaz peynir) on the fatty acid and volatile content. Int. J. Dairy Technol..

[28] Ferreira I.M.P.L.V.O., Pinho O., Sampaio P. (2009). Volatile fraction of DOP “Castelo Branco” cheese: Influence of breed. Food Chem..

[29] Zeng H., Wang Y., Han H., Cao Y., Wang B. (2022). Changes in Key Aroma Compounds and Esterase Activity of Monascus-Fermented Cheese across a 30-Day Ripening Period. Foods.

[30] García-Cayuela T., Gómez de Cadiñanos L.P., Peláez C., Requena T. (2012). Expression in Lactococcus lactis of functional genes related to amino acid catabolism and cheese aroma formation is influenced by branched chain amino acids. Int. J. Food Microbiol..

[31] Jiao J., Zheng Z., Liu Z., You C. (2021). Study of the Compositional, Microbiological, Biochemical, and Volatile Profile of Red-Veined Cheese, an Internal Monascus -Ripened Variety. Front. Nutr..

[32] Papaioannou G., Kosma I., Badeka A.V., Kontominas M.G. (2021). Profile of volatile compounds in dessert yogurts prepared from cow and goat milk, using different starter cultures and probiotics. Foods.

[33] Gatzias I.S., Karabagias I.K., Kontominas M.G., Badeka A.V. (2020). Geographical differentiation of feta cheese from northern Greece based on physicochemical parameters, volatile compounds and fatty acids. LWT.

[34] Hayaloglu A.A., Cakmakci S., Brechany E.Y., Deegan K.C., Mcsweeney P.L.H. (2007). Microbiology, Biochemistry, and Volatile Composition of Tulum Cheese Ripened in Goat’ s Skin or Plastic Bags. J. Dairy Sci..

[35] McSweeney P.L.H. (2004). Biochemistry of cheese ripening. Int. J. Dairy Technol..

[36] Topçu A., Saldamli I. (2006). Proteolytical, Chemical, Textural and Sensorial Changes During the Ripening of Turkish White Cheese Made of Pasteurized Cows’ Milk. Int. J. Food Prop..

[37] Lawrence R.C., Creamer L.K., Gilles J. (1987). Texture Development During Cheese Ripening. J. Dairy Sci..

[38] Hailu Y., Hansen E.B., Seifu E., Eshetu M., Petersen M.A., Lametsch R., Rattray F., Ipsen R. (2018). Rheological and sensory properties and aroma compounds formed during ripening of soft brined cheese made from camel milk. Int. Dairy J..

[39] Basiony M., Hassabo R. (2022). Composition and Quality of Low-Fat Halloumi Cheese Made Using Modified Starch as a Fat Replacer. Starch—Stärke.

[40] Ozbek C., Guzeler N. (2022). Effects of stabilisers in brine on soft white cheese quality parameters. Int. Dairy J..

[41] Wang D., Cheng F., Wang Y., Han J., Gao F., Tian J., Zhang K., Jin Y. (2022). The Changes Occurring in Proteins during Processing and Storage of Fermented Meat Products and Their Regulation by Lactic Acid Bacteria. Foods.

[42] Hamdy A.M., Ahmed M.E., Mehta D., Elfaruk S., Hammam A.R.A., El-derwy Y.M.A. (2021). Enhancement of low-fat Feta cheese characteristics using probiotic bacteria. Food Sci. Nutr..

