# Supplementary material for: Evaluation of a New Bulk Packaging Container for the Ripening of Feta Cheese
Source: Foods. 2023 May 28;12(11):2176. doi: 10.3390/foods12112176 (PMC10252536; doi:10.3390/foods12112176)
Supplement: Supplementary file 1 [file foods-12-02176-s001.zip › foods-2391914-supplementary.pdf]

Supplementary Material

Evaluation of a New Bulk Packaging Container for the Ripening of Feta Cheese

Panagiotis Thodis[1], Ioanna S. Kosma[1], Konstantinos Nesseris[2], Anastasia V. Badeka[1*], Michael G. Kontominas[1*]

1 Laboratory of Food Chemistry, Department of Chemistry, University of Ioannina, 45110, Ioannina, Greece.

2 DODONI S.A. Agricultural Dairy Industry of Epirus, 1 Tagmatarchi Kostaki, Eleousa 45500, Ioannina, Greece.

* Correspondence: mkontomi@uoi.gr; abadeka@uoi.gr

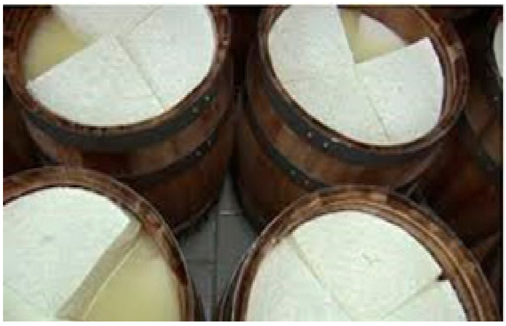

(a)

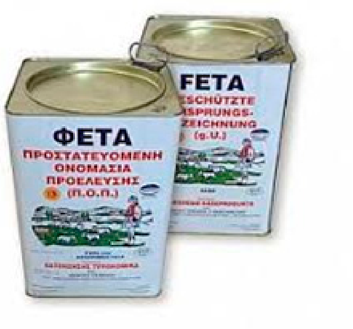

(b)

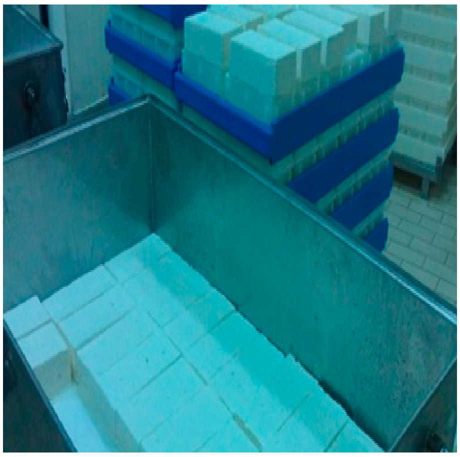

(c)

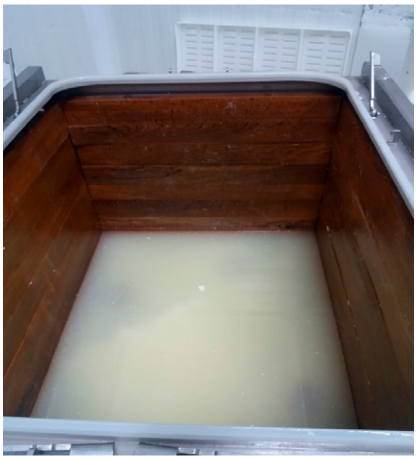

(d)

Figure S1: (a) wooden barrel, (b) tin can, (c) stainless steel tank and (d) stainless steel tank lined with wood for the ripening/bulk packaging of feta cheese.
